# Knowledge, attitudes and practices of faculty on mentorship: an exploratory interpretivist study at a sub-Saharan African medical school

**DOI:** 10.1186/s12909-020-02101-9

**Published:** 2020-06-15

**Authors:** Aloysius G. Mubuuke, Scovia N. Mbalinda, Ian G. Munabi, David Kateete, Robert B. Opoka, Sarah Kiguli

**Affiliations:** 1 School of Medicine, Makerere University, Kampala, Uganda; 2 School of Health Sciences, Makerere University, Kampala, Uganda; 3 School of Biomedical Sciences, Makerere University, Kampala, Uganda

**Keywords:** Mentorship, Faculty

## Abstract

**Background:**

Mentorship has become a routine part of undergraduate training in health professions education. Although many health professions training institutions have successfully incorporated faculty-student mentorship in their formal training, many others especially in Sub-Saharan Africa have not fully embraced this. Institutionalized mentorship programmes are effective methods of enhancing student learning experiences. Faculty, who are the mentors have an active role to play in driving the mentorship agenda and ensure that students benefit from this important activity. The aim of this study was to explore the knowledge, attitudes and practices of faculty about student mentorship at Makerere University College of Health Sciences.

**Methods:**

It was an exploratory qualitative study using interviewer-administered semi-structured questionnaires. The study participants included faculty at Makerere University College of Health Sciences. Thematic analysis was used to analyse the data using pre-determined themes.

**Results:**

Four themes were identified: 1) Knowledge of mentorship, 2) Attitude towards mentorship, 3) Practice of mentorship and 4) Improving the mentorship process. Majority of the faculty reported being less knowledgeable on mentorship regardless of seniority. The level of knowledge seemed to influence the practice of mentorship. Despite the observed knowledge gap, all faculty demonstrated a positive attitude to participate in mentoring.

**Conclusion:**

Faculty demonstrated a positive attitude towards mentorship despite the knowledge gap of mentorship identified. Continuous faculty development in mentorship as well as using peer mentorship were identified as key in sustaining the mentorship programme.

## Background

Mentorship has been defined as a process in which a more experienced person assists a less experienced person through their career growth and progression [1]. The benefit of mentorship has been a subject of considerable research in health professions education [2, 3]. This body of knowledge, gathered across a continuum of professional domains, indicates that mentorship has a significant outcome on learner success, satisfaction, and professional development [4]. Within the realm of higher education, and specifically in health professions education, mentorship has been associated with academic and social success across disciplines and through the learning process [5]. Focused and strong mentorship has been linked to enhanced mentee productivity, self-efficacy, career satisfaction and a sense of belonging and support [6, 7]. Learners who participate in active mentorship relationships are more likely to persist in their academics and make positive academic and social decisions [8, 9], with positive mentoring being cited as the most important factor in completion of studies [10]. Moreover, mentored students and their faculty mentors are more likely to publish their research than counterparts who are not mentored, thus contributing to the institutional profile [11, 12].

Various mentorship domains, such as socio-emotional (e.g., psycho-social support) and instrumental (e.g., research task support, learning task support) mentoring, have been positively associated with students’ identity as learners, their sense of belonging, and their confidence to function as future professionals [13, 14]. These factors have also been associated with increased interest and commitment to learning and engaging in research [15–17]. Undergraduate student learning and research experiences have also been shown to effectively increase their interest, motivation, and preparedness for professional practice with a positive mentoring relationship often cited as a key element in these outcomes [18–20]. The quality of mentorship relationships, largely influenced by faculty mentors, has been associated with students’ persistence to learn and become better, with mentoring directly or indirectly impacting on learner performance [21, 22].

At Makerere University College of Health Sciences from where this study was conducted, efforts have been made in the past to introduce mentorship for undergraduate students, but with variable success. Not much empirical evaluation of the mentorship programme has taken place. Specifically, faculty knowledge, attitudes and practices regarding mentorship has not been deeply explored.

There is also less published literature from the Sub-Saharan African context with low-resource settings where the number of faculty mentors is limited, yet with increasing student numbers exploring the knowledge, attitudes and practices of faculty in as far as mentoring students is concerned. The theoretical context within which this study was conducted related to faculty development through initially obtaining baseline data on current knowledge, attitudes and practices of faculty regarding mentorship. This would thus inform the organization of training programmes on mentorship and subsequently offer a significant starting point in as far as starting a sustainable mentorship programme is concerned. Two studies have been previously conducted in Uganda in relation to mentorship [14, 15]. However, one of these studies explored the subject of mentorship focusing on status of mentorship practices at Makerere University College of Health Sciences [14], without exploring faculty knowledge and attitudes towards these mentorship practices. The second study largely explored the concept of doctoral supervision from doctoral students and their supervisors from the same institution [15], relating it to mentorship. However, mentorship is different from supervision. Therefore, the purpose of this study was to explore the knowledge, attitudes and practices of faculty on mentorship building upon these previous studies so as to generate formative baseline data that can inform the establishment of more effective approaches to sustaining mentorship programs especially in institutions where mentorship has not yet taken strong root. The aim of the study was not to interrogate and establish or even compare mentorship approaches across different contexts, but rather to establish baseline information regarding the knowledge, attitudes and practices of faculty mentors in a human resource-constrained institution. Findings from the study can assist other institutions in a similar context to eventually better organize and train faculty adequately, before starting formal mentorship programmes.

## Methods

### Study design

It was an exploratory qualitative study in which semi-structured individual interviews were conducted with faculty at Makerere University College of Health Sciences (MaKCHS). Individual interviews assist in collecting insightful descriptions from participants. Open-ended questions were used and responses tape-recorded. All participants in this study were faculty at MaKCHS. There were no restrictions to faculty seniority. Faculty who did not consent to participate were excluded from the study.

### Context

The study was conducted at Makerere University College of Health Sciences (MaKCHS) in Uganda, the oldest health professions training institution in the East African region. The purpose of introducing mentoring for undergraduate students was basically to create a process where students can periodically interact with the more experienced faculty mentors and learn from these mentors not only knowledge and skills, but also other generic competencies such as communication, time management, interpersonal skills, social skills and many more. It was also aimed at creating a process where students can quickly get assistance regarding their psycho-social challenges from the more experienced faculty beyond academics there by facilitating the process of nurturing a holistic health care professional. All newly recruited faculty undergo an induction session during which mentorship is one of the aspects talked about. This session lasts about 1 h and its role is to orient new faculty into the institution. In addition, the institution has mentorship guidelines that were drafted by a select team of faculty with competency in mentorship. It is assumed that such guidelines would be accessible to all faculty. However, there are no formal structured and institutionalized periodic refresher mentorship training sessions that follow thereafter. The induction mentorship training generally follows the general principles of mentorship reported in literature [22]. Faculty are introduced to the meaning of mentorship and it is emphasized that it is a developmental relationship in which they are expected to assist students to cope and transition through their stay in the institution with a focus on not only academic progress, but also other social skills. Therefore, as mentors, the faculty are reminded that they should guide students outside academic work through any social challenges, psychological challenges and helping them identify resourceful people that can assist them in their development through medical school. The faculty are also introduced to roles of a mentor as a guide, teacher, coach and counsellor who should actively listen and provide constructive feedback that can assist students to develop their full potential. This thus largely constitutes the meaning of mentorship within the context of the institution where this study took place through which the faculty are inducted.

Students from first year are randomly assigned faculty mentors with whom they are expected to forge a fruitful and rewarding mentorship relationship. However, the mentorship process has met some challenges such as increasing student numbers as well as students reporting that they do not receive adequate attention from their assigned faculty mentors. This could be possibly due to factors such as limited competency of the faculty to effectively mentor students.

### Study participants

Thirty-seven (37) faculty participated in the study through purposive-convenience sampling. The College of Health Sciences conducts an induction mentorship training session for new faculty. The participants selected were those participants that had at least undergone this induction training session on mentorship. Newly recruited faculty at the time of the study were not selected owing to their presumed limited experience with mentoring. This sample size provided enough data saturation. The participant responses had become repetitive and redundant with the 32nd participant and no new ideas were coming up. However, five more interviews were conducted to confirm data saturation. These responses provided adequate depth which is key in focused exploratory qualitative studies like this study rather than dwelling on the breadth of the sample size that provide similar responses even after data saturation.

### Data collection

Data was collected using semi-structured individual interviews conducted with faculty at MaKCHS (*Additional File 1*). The interview guide used was developed particularly for this study and was first pre-tested with two faculty before commencing data collection. Open-ended questions were used to explore faculty knowledge, attitudes and practices towards mentorship. The language used to conduct the interviews was English because all participants were competent in English. The responses were audio-recorded and later transcribed. In addition, some field notes were written to back up the recorded responses. Each interview lasted somewhere between 45 min to 1 h.

### Data analysis

Thematic analysis, a valued method for analyzing qualitative data was used. The analysis process commenced immediately after the first interview. The process of open coding was adopted. This process involved reading the transcribed interviews and assigning raw codes. Transcribed raw data was constantly proof-read against the audio-taped interviews for clarity. The initial codes generated were read and compared and codes of similar meaning were grouped into categories. These categories were also read and compared with each other. Related categories were also grouped into larger themes. These themes summarized the meaning of the data which addressed the purpose of the study. The emergent themes and related categories were sent back to the participants as a check measure to ensure that their responses had been represented. Out of the 37 participants, 34 responded and were satisfied with the themes. Three participants never responded back despite several reminders. The eventual themes were presented along with representative participant responses to provide context.

### Ethical considerations

Approval to conduct this study was granted by the Research and Ethics Committee, School of Medicine, Makerere University (Protocol No: REC REF 2019–007). Written informed consent was also obtained from each study participant prior to conducting the interviews. Confidentiality, autonomy, respects and dignity of all the participants was strictly observed throughout the study. In addition, participants were assured of their rights to decline participating in the study and also not to answer questions they felt uncomfortable with. The participants were also assured that there will be no harm, prejudice, malice or any form of danger should they wish not to participate in the study.

## Results

### Demographic information

Thirty-seven (37) faculty participated in the study, 67.6% (*n* = 25) were male and 32.4% (*n* = 12) were female. The faculty had a range of teaching experiences at MaKCHS. 8.1% (*n* = 3) were Professors, and 13.5% (*n* = 5) were Associate Professors, 24.3% (*n* = 9) were Senior Lecturers, 32.4% (*n* = 12) were Lecturers and 21.6% (*n* = 8) were Assistant Lecturers. Although all the 37 participating faculty had heard about mentorship, less than half (37.8%, *n* = 14) had received some more form of training in mentorship beyond the induction training session participated in as newly recruited faculty. Those that had received some more training on mentorship reported varied sources ranging from short meetings about mentorship, training workshops and conference attendance on mentorship sessions. Slightly more than half (59.6%, *n* = 22) of the participants had previously acted as either formal or informal mentors through either formal appointment as mentors or as informal requests from students to be their mentors. When asked about their self-reported competency in mentorship, less than half of the participants (35.1%, *n* = 13) rated themselves as being competent, 27% (*n* = 10) of them were neutral and the rest of the participating faculty (37.8%, *n* = 14) rated themselves as being less competent. When asked whether they need more training in student mentorship, all participating faculty (100%) reported that they would welcome more training in mentorship in order for them to effectively mentor students.

### Themes

Four key themes emerged from the data. These included: 1) Knowledge of mentorship, 2) Attitude towards mentorship, 3) Practices of mentorship and 4) Improving the mentorship process.

### Knowledge of mentorship

From the findings in this study, it generally appeared like the faculty had less knowledge about mentorship and there was need to boost their formal knowledge of mentorship. The following responses reflected this theme.

“*I have always heard about mentorship from my colleagues and students as well. I was even allocated students to mentor … however, I did not know how to start and what steps to follow as I mentor the students … .as a result, the relationship never really worked out … .*”

“*I have personally read about mentorship and tried to practice it. However, I am not sure if what am practising is really mentorship or not. I got assigned students to mentor, but got stuck somewhere because I did not figure out how this whole mentorship thing will go besides having many students allocated to me. Perhaps we need some training first before students can be thrown on to us … ..I also got into a fix whether as a research supervisor, I was indirectly mentoring.*”

The few faculty who had some knowledge about mentorship had received such knowledge through short meetings and short training workshops.

“*The College has previously organized some meetings where mentorship was talked about. Not all faculty attend these meetings. However, such meetings are also short and I myself would have preferred continued refresher training in mentorship. Mentorship is an important process in developing our students and we are required to be good mentors. However, am not sure if am good mentor … I need more training on my roles as a mentor and what I expect from students and vice versa from this mentorship relationships.*”

The responses above therefore indicate that while many of the faculty who participated in the study had less knowledge about mentorship, a few had some level of knowledge which they had received through short organized meetings on mentorship.

### Attitude towards mentorship

Findings from the study generally reflected a positive attitude of faculty towards mentorship. There was a common denominator of faculty willingness to participate in mentoring students provided they have been given the skills to become effective mentors. This was reflected in the following responses:

“*I am more than ready and interested in mentoring students provided I have the skills. Right now, I am just learning on job sort of because I was also not mentored … ..if the College can organize some training sessions and we get the skills, I am happy to mentor my students effectively.*”

“*Mentorship is not bad and am sure many staff here in the College would like to be good mentors. The challenge is that we ourselves probably do not know how to mentor very well because no one taught us. It is the same with teaching since no one taught us how to teach well. So if I am trained for example in how to be a good mentor and what mentorship entails, I am willing and ready to be a good mentor.*”

“*I always interact with undergraduate students on the ward and teach them as long as they find me there. Is that part of mentorship? I am not sure … .but if that is part of mentorship then I can say I mentor. If not, am willing to learn more about effective mentorship so that my students can achieve maximum benefit.*”

The positive attitude to get involved in student mentorship seemed to also be influenced by the current institutional willingness to form a formal mentorship structure and actively involve faculty in the student mentorship process

“*The College has the will to develop mentorship in this institution and I remember there was a mentorship committee sometime back that even developed some form of guidelines on mentorship. The problem is that these remained on paper … .if we can revive this, I am more than ready to participate again in student mentorship because it is an important process and this is where the world is going … .*”

### Practice of mentorship

There were mixed findings regarding the practice of mentorship by faculty. Practice in this context would relate to faculty actively getting involved in mentoring students and ensure that these mentorship relationships succeed. Some of the faculty reported to have participated in some formal mentorship of students allocated to them as reflected below.

“*I remember sometime back I was allocated some undergraduate students to mentor by the College coordination office. It was good at the beginning, but later I lost touch with the students. I do not know whether I was a bad a bad mentor and students decided to run away … .*”

“*I have been a mentor before for students when they rotate in my department and these have been allocated to me by my Head of Department. I tried to give them my time … ..many have succeeded and some of them keep consulting me on certain issues up to now … .*”

Some of the other faculty seemed to have initiated informal mentorship relationships with the students within their respective departments. The following responses represent this observation.

“*I have tried to identify students who are interested in working with me and encouraged them to consult on a range of issues including academics, challenges and any support that I might give them to make their experience in the department better. Would you call this mentorship...I am not very sure.*”

“*I and a few colleagues in our research labs have always worked with students especially those interested in pursuing a research career. In the process, they have acquired key research skills … ..nobody assigned these students to us but we just identify them and we supervise them as they o lab work. This can be a form of mentorship.*”

Further interrogation of the findings revealed that perhaps the limited knowledge on what mentorship entails might have had an influence to the reported practices of some faculty. For example, all faculty that participated in the study had interacted with students. However, some of them were not sure as to whether this interaction meant they were engaged in mentorship or not. The following responses contextualize this thinking:

“*I have always been with students in the tutorials and on the wards. During our engagement, I have taught them and encouraged them to ask questions if they needed clarification. I think I was mentoring them during these meetings … .maybe I can be corrected on this.*”

“*Many of us including myself have been supervising our students on the wards and in the labs during practicals and we always guide them to learn. Therefore as a supervisor, am indirectly mentoring them as well.*”

The above findings also indicate a key misconception between mentorship and supervision among the faculty.

### Improving the mentorship process

Another common thread that swept through the interviews with faculty related to improving the mentorship process. Through the faculty responses, suggestions for improving the mentorship process emerged. Key among these included: continuous training of faculty in mentorship, creating awareness among students about mentorship, use of peer mentorship where senior students do mentor junior students, institutionalizing mentorship such that there is a formal mentorship structure with specific guidelines for the faculty to follow, ensuring that all faculty participate by allocating them mentors in addition to those independently identified on their own and conducting periodic evaluation and quality assurance checks to get feedback from faculty and students so as to continuously improve the mentorship process. The following responses reflect some of the above suggestions.

“*I think the most important thing is continuous training of us the teachers about mentorship. Many of us were neither formally mentored nor formally trained in mentorship and therefore cannot apply it to our students effectively. We need training of faculty in effective mentorship.*”

“*There is need to sensitize students about mentorship as well. I would thus recommend may be meetings with students to talk to them about mentorship before being allocated mentors … .in addition, we are few as faculty and so training senior students to act as mentors to junior students can also be explored*”

“*Institutional buy-in along with mentorship guidelines are key aspects I would advise that can improve our mentoring practices. If mentorship guidelines do exist in this College, I have not seen them yet, if not, guidelines need to be drafted by experts so that they can assist us to effectively mentor our students.*”

The responses above relate to key suggestions for improving the mentorship process, the key being continued training and use of guidelines availed to faculty.

## Discussion

The purpose of this study was to explore the knowledge, attitudes and practices of mentorship by faculty at Makerere University, College of Health Sciences. The study adopted a faculty development theoretical context with collection of baseline data on knowledge, attitudes and practices of faculty on mentorship seen as the initial step towards informing targeted training mentorship training. This would potentially result into strengthened faculty capacity to sustain a mentorship programme. Findings from the study reflected a blend of experience of faculty from the lowest to the highest ranks. However, this trajectory of various academic ranks did not reflect adequate knowledge of mentorship much as many of these faculty staff had been involved in teaching students for some time. For example, more than half of the faculty had not received formal training in mentorship and many reported as being less competent in mentorship. This is perhaps attributable to the fact there is no structured and institutionalized periodic formal mentorship training programme for faculty beyond the initial induction session in which mentorship is talked about which lasts for only a short period of time. Besides, the induction programme for new faculty does not only focus on mentorship, but many other aspects expected of them.. In addition, it can also be an indication that being a senior staff does not necessarily make one a good mentor without formal training in mentorship, an observation that has been previously reported [6].

With no structured and periodic training in mentorship, the outcome of the mentorship relationships may not effectively benefit the learners [7]. Some of the faculty who had some mentorship knowledge reported having obtained it from short meetings and conferences with themes on mentorship. It is true that the institution may sometimes organize mentorship meetings to faculty and increase their knowledge on mentorship. However, such meetings are not formally structured within the institutional programmes, and are merely sensitization meetings to create awareness rather than focused mentorship trainings. In addition, it is not clear as to whether such short meetings do influence actual mentorship skills and practices of the faculty to benefit their learners. Some of the faculty who attended these meetings and thus reported having attained some knowledge on mentorship could also arguably have been influenced by illusory superiority due to cognitive bias because this knowledge seemed not to necessarily translate into good mentorship practices according to our observations from faculty responses. It has been reported that knowledge of mentorship should translate into actual skills to improve the mentorship experiences of student [11].

There is a possibility that mentorship training may be driven by donor funds/projects and this may not be sustainable. With no formal instutionalized mentorship training of faculty, it is thus not surprising that many of them were less knowledgeable in mentorship. This observation may have key implications for the institution and many other institutions that have adopted or are thinking of adopting formal mentorship programmes for their students. The consideration of training senior students to act as mentors to their fellow junior students also needs to be thought about amidst the shortage of faculty mentors. The senior students could potentially bridge the gap of limited faculty mentors. We hereby do recommend a model of mentorship training whereby not only faculty, but also senior students are trained in mentorship. Senior students could be post-graduate students or even undergraduate students in 4th or 5th year. These students can effectively mentor junior students if trained. Such a mentorship training model is one way of sustaining the mentorship programme in an institution with limited faculty mentors.. The idea of continuous training should be considered in this model because of the inevitable turn-over of faculty and students as some leave and new ones come on board. However, we do strongly advise that using senior students as mentors is fundamentally different from using experienced faculty. Therefore, if senior students are to be utilized as mentors, a clear role for them needs to be defined in the mentorship relationship cognizant of their time since they also have their own learning activities to attend to. Careful considerations must be taken not to load students with mentorship responsibilities and they need to be significantly trained before assigning them junior students and continuously monitored and evaluated during the mentorship processes. The mentees of such senior students should be given opportunity to also occasionally interact with experienced faculty mentors. One off meetings to create awareness about mentorship may not be adequate for faculty to grasp various aspects of effective mentorship. We did not observe significant variations in the knowledge levels among the various faculty cadres. This means that even some senior faculty were as less knowledgeable in mentorship as the junior faculty. Having more experience in teaching and interacting with students may thus not necessarily translate into being a good mentor to students [12].

Despite the reported knowledge gap about mentorship, all faculty who participated in the study generally demonstrated a positive attitude and willingness towards mentoring students. This intrinsic faculty motivation to engage in student mentorship should not be ignored, and it could be an important entry point by the institutional leadership. The explanation for the observed positive attitude is not clear-cut. However, such positive attitude expressed could perhaps be due to the fact that all faculty do interact with students and participate in teaching and learning. Therefore, we hypothesize that majority of the faculty are probably interested in the growth of their students. This may explain their willingness to train and become more effective mentors.

The faculty practices of being actively engaged in mentorship revealed mixed findings. For example, whilst some faculty reported being involved in some formal mentorship of students allocated to them, others were not sure if they were mentoring students when interacting with them. Yet others were not involved in mentorship at all. It has been reported that limited knowledge of mentorship can potentially influence the practice of engaging into mentoring others [13]. The deficit in knowledge of faculty regarding mentorship observed in this study arguably seems to have influenced their practices of mentorship. The fact that some faculty did not know as to whether they were mentoring or not when interacting with students deserves urgent attention. To problematize this further, all faculty that participated had at least undergone an induction session on mentorship and the College of Health Sciences has well documented mentorship guidelines. In addition, some seemed to create tension between mentorship and supervision. This points to two things. First, it is almost definite as demonstrated in this study that perhaps a single induction session for new faculty is not adequate. There is need to plan for periodic faculty development refresher training sessions if a strong mentorship culture is to be achieved. Second, the fact that some faculty had no clear knowledge on mentorship yet they had attended an induction session on mentorship and the institution has mentorship guidelines highlights this as an urgent issue to consider by the institution. The one session may not be adequate to synthesize what mentorship actually is. However, it also points to the fact that the institutional guidelines on mentorship are not known and are not availed to the faculty, but are simply kept where they cannot be accessed. As a first step, such mentorship guidelines need to be availed to the faculty during and after mentorship training sessions for reference. This can then be augmented by the periodic refresher training sessions on mentorship. In addition, mentorship and supervision are two different concepts. Though one can be both a mentor and a supervisor, one can be a supervisor without necessarily being a mentor [21]. There is greater need to deconstruct mentorship and supervision and create awareness among both faculty and students of how mentorship differs from supervision. We do believe that more focused and layered training of faculty would improve the situation by not only increasing knowledge of faculty, but also influencing their practices to engage in effective mentorship. With a positive attitude of faculty, this is likely to improve the mentorship experiences of the learners.

Key suggestions to improve mentorship have been reported in literature such as training of faculty, sensitization and formulation of mentorship guidelines [3]. Some of these suggestions were in resonance with findings from this study. Indeed, in a previous study by Nakanjako et al. [14], training of faculty in mentorship was also suggested. This means that since that time, not much has been achieved by the institution. There is thus need to implement and operationalize the mentorship guidelines already in place. Although, the institution where this study took place may perhaps have had some draft mentorship guidelines, these have largely been less disseminated and less operationalized to drive and standardize the mentorship process. With constraints of limited numbers of faculty mentors vis-à-vis the increasing student numbers, we do recommend that the mentorship guidelines need to be highly structured to make them feasible and easy to implement. There is also need to disseminate the formulated guidelines in a participatory approach to allow faculty have their in-put, which is likely to increase their acceptability.

Training of faculty on how to utilize the mentorship guidelines is essential, an observation that has been previously reported [14]. In addition to training faculty, sensitization of students about the benefits of mentorship is also important. Allocating mentors to students is a welcome move, however, for the mentorship relationships to effectively function, continued training and periodic quality monitoring and evaluation of the mentorship programme is paramount, another key implication from the study. Beyond training and availing guidelines, we do strongly advocate for the creation of monitoring and evaluation activities of the mentorship processes as a means of quality control. This can also assist in identifying gaps where improvement is needed. The observation that faculty had a positive attitude towards mentoring despite the less knowledge observed is a strength of this study. This could be an entry point in strengthening institutional mentorship when faculty are willing to actively participate. In addition, the mentorship training model suggested where both faculty and senior students are trained in mentorship could be an effective way of ensuring sustainability. However, it should also be remembered that one critical element of a successful mentoring relationship is the aspect of time for both mentor and mentee. There is need to factor in the issue of time such that there is time left for both parties to engage in other activities. This should be taken into account when designing the mentorship programme. One way of addressing this is to emphasize to both mentors and mentees during training that mentorship contracts need to be drawn in which mentorship meetings occur at only agreed upon times. Despite the useful information generated from this study, there are some limitations. The study adopted an exploratory qualitative approach involving non-probability sampling in a single institution. This could thus limit the generalizability of the findings. In addition, the study only employed interviews with faculty mentors, another limitation. Perhaps including a simple survey or even a knowledge based test on mentorship would have added rigor to our findings. Furthermore, having separate interview guides that put into consideration the different levels and experiences of faculty mentors would be another advantage which was not considered in this study. Nonetheless, the study provides key insights and baseline information on faculty experiences and practices of mentorship that may be transferable to many other settings and which can be utilized in future survey based studies that involve large numbers of faculty.

## Conclusion

The faculty who participated in this study were a representation of a range of teaching experience including both senior and junior faculty. The study demonstrated that majority of them had less knowledge of mentorship regardless of seniority. The few faculty that had some knowledge of mentorship attained it largely through meetings. Although the limited knowledge on mentorship seemed to influence the practice of mentoring students, all the faculty expressed a positive attitude and willingness to mentor students. Continuous faculty development in mentorship, involvement of senior students in mentorship training and formulation of structured mentorship guidelines along with quality monitoring are likely to improve the mentorship processes and ensure sustainability in resource-constrained institutions. Ultimately, institutions need to map out context-specific mentorship programmes that can work in that particular area rather than relying solely on programmes developed elsewhere.

## Supplementary information

**Additional file 1: Interview guide.** This provides the details of the questions that were asked the participants during the individual interviews.

## Abbreviations

MakCHS: Makerere University College of Health Sciences
