# Supplementary material for: Knowledge, attitudes and practices of faculty on mentorship: an exploratory interpretivist study at a sub-Saharan African medical school
Source: BMC Med Educ. 2020 Jun 15;20:192. doi: 10.1186/s12909-020-02101-9 (PMC7294640; doi:10.1186/s12909-020-02101-9)
Supplement: Supplementary file 1 — Additional file 1: Interview guide. This provides the details of the questions that were asked the participants during the individual interviews. [file 12909_2020_2101_MOESM1_ESM.docx]

**ADDITIONAL FILE 1**

**INTERVIEW GUIDE**

I politely greet the participant and introduce myself to the participant and also request him/her to introduce himself/herself. I explain the purpose of the study and how it will be beneficial to the institution and our students` experiences as well. I then explain that participation is voluntary and the participant is free to decline participation or even terminate the interview if they don’t feel comfortable. I also explain that there are no risks of any form from the study. I then administer the consent form and the interview commences as follows:

**1. Briefly share your past experience with mentorship either with someone whom you regarded as a mentor or someone that you personally thought you were mentoring**? (*This is meant to break the ice and set the mood of the interview. Prompt the participant to just generally share any past exposure to mentorship without going into details*).

**2. Okay, please share with us your understanding of what mentorship of student means within the context of your institution?** (*Prompt the participant to share his/her knowledge of student mentorship broadly and then narrow the discussion to within the context of the College of Health Sciences*).

**3. Could you please share with us how learned about the concept of mentorship including what is involved?**

**Probes**: (*Principles of mentorship*)-*Ask about what entails mentorship broadly and then* within the context of the institution

(*Roles of a mentor*)-*Probe for participant to highlight some roles of the mentor*

(*Roles of a mentee*)-*Probe for participant to highlight some roles of a mentee*

**4. Could you please share your views and opinions regarding mentoring our students?**

**Probes**: -*Do you think it is good and in what ways?*

*- Would you be willing to participate as a mentor?*

**5.** **Please share with us your past experiences as a mentor in this institution**

**Probes**: -*Have you been a mentor to some students?*

*-Are you still mentoring some students?*

*-Do you have students consulting you to be their mentor?*

*-Have you personally reached out to some students to mentor them?*

*-How does some feedback prevent you from learning?*

**6.** **Do you have any other comments please?**
